# The new SRS/FSRT technique HyperArc for benign brain lesions: a dosimetric analysis

**DOI:** 10.1038/s41598-021-00381-9

**Published:** 2021-10-26

**Authors:** Hsiu-Wen Ho, Ching-Chieh Yang, Hsiu-Man Lin, Hsiao-Yun Chen, Chun-Chiao Huang, Shih-Chang Wang, Yu-Wei Lin

**Affiliations:** 1 Department of Radiation Oncology, Chi Mei Medical Center, Tainan, Taiwan; 2 Department of Pharmacy, Chia-Nan University of Pharmacy and Science, Tainan, Taiwan; 3 Department of Radiation Oncology, National Cheng Kung University Hospital, Tainan, Taiwan; 4 Department of Radiation Oncology, Kaohsiung Veterans General Hospital, No.386, Dazhong 1st Rd., Zuoying Dist., Kaohsiung City, 813414 Taiwan

**Keywords:** CNS cancer, Biological physics

## Abstract

To evaluate the potential benefit of HyperArc (HA) fractionated stereotactic radiotherapy (FSRT) for the benign brain lesion. Sixteen patients with a single deep-seated, centrally located benign brain lesion treated by CyberKnife (CK, G4 cone-based model) were enrolled. Treatment plans for HA with two different optimization algorithms (SRS NTO and ALDO) and coplanar RapidArc (RA) were generated for each patient to meet the corresponding treatment plan criteria. These four FSRT treatment plans were divided into two groups—the homogeneous delivery group (HA-SRS NTO and coplanar RA) and the inhomogeneous delivery group (HA-ALDO and cone-based CK)—to compare for dosimetric outcomes. For homogeneous delivery, the brain V5, V12, and V24 and the mean brainstem dose were significantly lower with the HA-SRS NTO plans than with the coplanar RA plans. The conformity index, high and intermediate dose spillage, and gradient radius were significantly better with the HA-SRS NTO plans than with the coplanar RA plans. For inhomogeneous delivery, the HA-ALDO exhibited superior PTV coverage levels to the cone-based CK plans. Almost all the doses delivered to organs at risk and dose distribution metrics were significantly better with the HA-ALDO plans than with the cone-based CK plans. Good dosimetric distribution makes HA an attractive FSRT technique for the treatment of benign brain lesions.

## Introduction

Stereotactic radiosurgery (SRS) or fractionated stereotactic radiotherapy (FSRT) has been demonstrated over the past decades to be a valuable modality in the management of patients with benign intracranial tumors^1^. The rate of local control after either SRS or FSRT is 80–95% at 5–10 years, with a low incidence of long-term toxicity^2–5^. Treating benign intracranial lesions with FSRT is advantageous because it provides a theoretical radiobiological benefit and a lower rate of symptomatic edema than single-fraction SRS^1,6–8^. In general, FSRT is delivered via a linear accelerator (Linac) with either cone-based or multileaf collimator (MLC)-controlled delivery. The CyberKnife (CK, Accuray Inc., Sunnyvale, CA, USA) stereotactic radiotherapy system is a frameless, image-guided method for delivering radiation to a precisely targeted volume using multiple noncoplanar beam angles with a steep surrounding-dose gradient^9,10^.

Recent radiotherapy developments, such as volumetric modulated arc therapy (VMAT), have made it feasible to apply such techniques to treat large, complicated, or multiple brain lesions through multiple arcs. RapidArc (RA, Varian Medical System, Palo Alto, CA, USA) is an isocentric coplanar/noncoplanar VMAT technique that can deliver highly conformal, intensity-modulated radiation doses in a single rotation or multiple rotations of the linear accelerator gantry^11^. RA enables higher-quality treatment plans than multifield intensity modulated radiation therapy (IMRT) while reducing the treatment time per fraction in the FSRT setting^12^. HyperArc (HA, Varian Medical System) is a new isocentric VMAT technique explicitly developed for noncoplanar, MLC-based stereotactic radiotherapy with automated optimizations and delivery^13,14^. The current study aimed to evaluate the feasibility of the HA technique by comparing its dosimetric measurements with those of other FSRT techniques, coplanar RA and cone-based CK (G4 model), for a single deep-seated, centrally located benign brain lesions.

## Methods

### Ethics approval and consent to participate

The Institutional Review Board approved this study at Chi Mei Medical Center. The institutional review board waived the participants’ need for written informed consent because this was an electronic treatment plan-analysis study. This study was performed following the relevant guidelines and regulations.

### Study groups

Sixteen patients who had undergone CK from 2018 to 2021 for a single deep-seated, centrally located benign brain lesions were enrolled. The 16 delivered CK treatment plans were replanned using for the corresponding coplanar RA and HA plans. The characteristics of the 16 benign brain lesion patients are shown in Table 1. In this study, two groups of FSRT treatment plans are the homogeneous and inhomogeneous delivery groups. Traditionally, the characteristics of the MLC-based Linac treatment plan is a more homogeneous dose distribution^15,16^. The homogeneous delivery group (keeping the maximal dose of the treatment plan less than 110%) was generated for each patient to meet the same corresponding CK treatment plan criteria by using HA with SRS normal tissue objective (SRS NTO) optimization algorithm (HA-S) and coplanar RA techniques. The inhomogeneous delivery group was designed to increase the dose of PTV and emulate the inhomogeneous CK isodose distributions (the maximal dose of the treatment plan between 111 to 120%) by using HA with the automatic lower dose objective^17^ (ALDO) optimization algorithm (HA-A).

**Table 1:** Characteristics of benign brain lesion patients.

Parameters	Patient
Patient number	16
**Disease**	
Pituitary adenoma	5
Meningioma	4
Cerebellopontine angle tumor	3
Cavernous malformation	1
Neurilemoma	1
Cival chodosarcoma	1
Arteriovenous malformation	1
**GTV (c.c.)**	
Median	5.25
SEM	0.83
Range	0.77–13.32
**PTV (c.c.)**	
Median	8.29
SEM	1.27
Range	0.77–19.52
Fractions	5
Prescription dose (Gy)	25

*CTV* clinical target volume, *PTV* planning target volume.

### Contouring and essential treatment plan criteria

The CTV was defined as the volume of the benign brain lesion. The PTV was extended from 0 to 2 mm from the boundaries of the CTV. The prescription dose of each of the sixteen enrolled patients was 25 Gy in 5 fractions. The details of the planning objectives for the targets and OARs are listed in Table 2. The coverage of clinical target volumes (CTVs) and planning target volumes (PTVs) was determined by the proportion of the CTV or PTV that received 100% of the prescription dose. A minimum of 95% of the prescription dose was assumed to cover 95% of the PTV after normalization to an 80% (or higher) isodose line. The OARs are the spinal cord, brainstem, optic pathways (including eyes, optic nerves, optic chiasma, and lens). No margin was added to a OAR to define a planning OAR volume (PRV). The priority of the treatment planning was sparing of OARs, followed by target coverage. Brain (including target volumes) Vx reflected the volume receiving greater than or equal to a dose of x Gy^18^.

**Table 2:** Planning objectives for the target and organs at risk.

Structures	Dose-volume constraints	Priority
PTV	Dmax ≤ 120% of the prescription dose	3
	95% of the prescription dose cover 95% of the PTV	2
Spinal cord	Dmax < 30 Gy	1
	V23 < 0.35 c.c.	
Brainstem	Dmax < 30 Gy	1
	V23 < 0.5 c.c.	
Optic pathway	Dmax < 25 Gy	1
	V23 < 0.2 c.c.	
Lens	Dmax < 4 Gy	3

*Dmax* the maximal point dose (0.0035 cc or less) of the structures, *V23* the volume of the organ at risk that receiving more than 23 Gy.

### CyberKnife treatment plans

The CK treatment plans of the sixteen patients who had been treated by CK (G4 cone-based model, Accuray Inc., Sunnyvale, CA, USA) were generated. In brief, all patients were immobilized in a supine position with a premolded U-frame mask. CT simulations were performed with 1-mm-thick slices at a resolution of 512 by 512 pixels. All treatment plans were generated with MultiPlan software (version 2.2.0, Accuray Inc.) with 6 MV flattening-filter-free photon beams using cone collimators. A nonisocentric beam arrangement was applied in all cases. The numbers of cones and beam nodes were arranged to optimize the target volume coverage and the sparing of OARs. A simplex optimization algorithm was used to optimize the MU production per beam. The radiation dose was calculated with the ray-tracing algorithm.

### HyperArc treatment plans

Computed tomography data sets and target/OAR contours from the sixteen enrolled patients were transferred from the CK system to the Eclipse treatment planning system (version 15.5, Varian Medical System Inc.). The corresponding HA plans were then generated according to the corresponding CK treatment plan’s dose prescription and OAR constraints. The HA plans were designed using 6 MV flattening-filter-free photon beams and a 1400 MU/min dose rate from Varian TrueBeam (Varian Medical System Inc.) equipped with a high-definition 120-leaf multileaf collimator (with a dynamic beam aperture and a spatial resolution of 2.5 mm leaf width × 32 pairs at the center, 5 mm width × 28 pairs in the peripheral leaves, and maximum static field size 40 cm × 22 cm). The Eclipse system automatically arranged arc fields: one full or half coplanar arc with a couch rotation of 0° and up to three partial noncoplanar arcs with couch rotations of 315°, 45°, and 90° (or 270°)^17,19^. The collimator angle and field size were optimized to maintain the coverage of the targets and reduce the radiation dose to OARs. The photon optimization algorithm for the homogeneous delivery group and the inhomogeneous delivery group was SRS NTO and ALDO (version 15.5.11, Varian Medical System Inc.). The calculation algorithms of Acuros XB (version 15.5.11, Varian Medical System Inc.) were applied for all cases.

### RapidArc treatment plans

The details of the RA treatment plans were similar to those of the HA treatment plans, as described above. The isocenter in the RA plan was set to be the same as in the HA plan. The two-coplanar-arc technique (counterclockwise rotation from 179° to 181° and clockwise rotation back to the starting position) was applied for all RA treatment plans. For all RA plans, the optimization algorithm and the calculation algorithm were the Photon Optimizer and Acuros XB algorithm. The normal tissue objective optimizer for RA was the automatic NTO (version 15.5.1, Varian Medical Systems).

### Plan evaluation statistics

The parameters used to evaluate the quality of the planned dose distributions for these FSRT plans (HA-S, coplanar RA, HA-A, and cone-based CK) were target coverage, sparing of OARs, and the main dosimetric parameters, including the point maximal dose (Dmax), which defined as 0.0035 c.c. or less, recommended by the report of AAPM Task Group 101^20^. For CTV and PTV, D2% was used to evaluate the near-maximum dose and D98% for evaluating the near-minimum dose^15^.

### Dosimetric parameters

The treatment plans were evaluated by comparing the dosimetric parameters derived from the DVHs for target coverage and sparing of OARs. The conformity index (CI) that previously described by Paddick^21^:

$$CI=\frac{{TV}_{{PIV}^{2}}}{TV \times PIV},$$

where TV_PIV_ is the target volume covered by the prescription isodose volume, TV is the target volume, and PIV is the prescription isodose volume. The higher the CI is, the more conformity the plan. A CI value of 1 stands for the ideal conformity.

The homogeneity index^15^ (HI) was determined as:

$$HI=\frac{{D}_{2\%}-{D}_{98\%}}{{D}_{50\%}}.$$

An HI of zero indicates that the absorbed-dose distribution is almost homogeneous.

### Dose gradient

The dose spillage has been introduced to reflect the dose falloff outside the target. The lower isodoses outside the PIV volume may cover significant amounts of normal tissues and can be responsible for complications, especially when the target is in proximity to critical structures. For estimation of dose falloff outside the target, the metrics evaluated include intermediate dose spillage and high dose spillage.

Intermediate dose spillage^22,23^ is calculated as:

$$Intermidate \,dose\, spillage= \frac{The\, volume\, receiving \,50\%\, of \,prescription\, dose}{PTV\, volume}.$$

For high dose spillage^24,25^ evaluation, volume of 105% isodose volume outside the PTV is estimated as a ratio of the volume of PTV.

$$High\, dose \,spillage= \frac{The\, volume\, of\, 105\%\, isodose\, volume\, outside\, the\, PTV}{PTV\, volume}.$$

The gradient radius, measured in cm, was calculated as the difference between the equivalent sphere radii of the volume of 50% of the prescription isodose curve and the prescription isodose volume^26^. A low gradient radius indicates a low dose spread outside the lesion and a sharp dose falloff.

$$Gradient\, radius=\sqrt[3]{\frac{3{V}_{50\%}}{4\pi }}-\sqrt[3]{\frac{3{V}_{100\%}}{4\pi }.}$$

### Statistical analysis

The dosimetric endpoints of the target volumes (CTV and PTV) and OARs, CIs, HI, intermediate and high dose spillage, gradient radius, and MUs were analyzed using the Wilcoxon signed-rank test (SPSS Statistics, Version 19, IBM, NY, USA). All tests were 2-tailed, with a *P*-value < 0.05 considered to be statistically significant.

### Ethics approval

The institutional review board waived the need for written informed consent from the participants because this was a retrospective electric treatment plan-review study.

## Results

**Figure 1:**
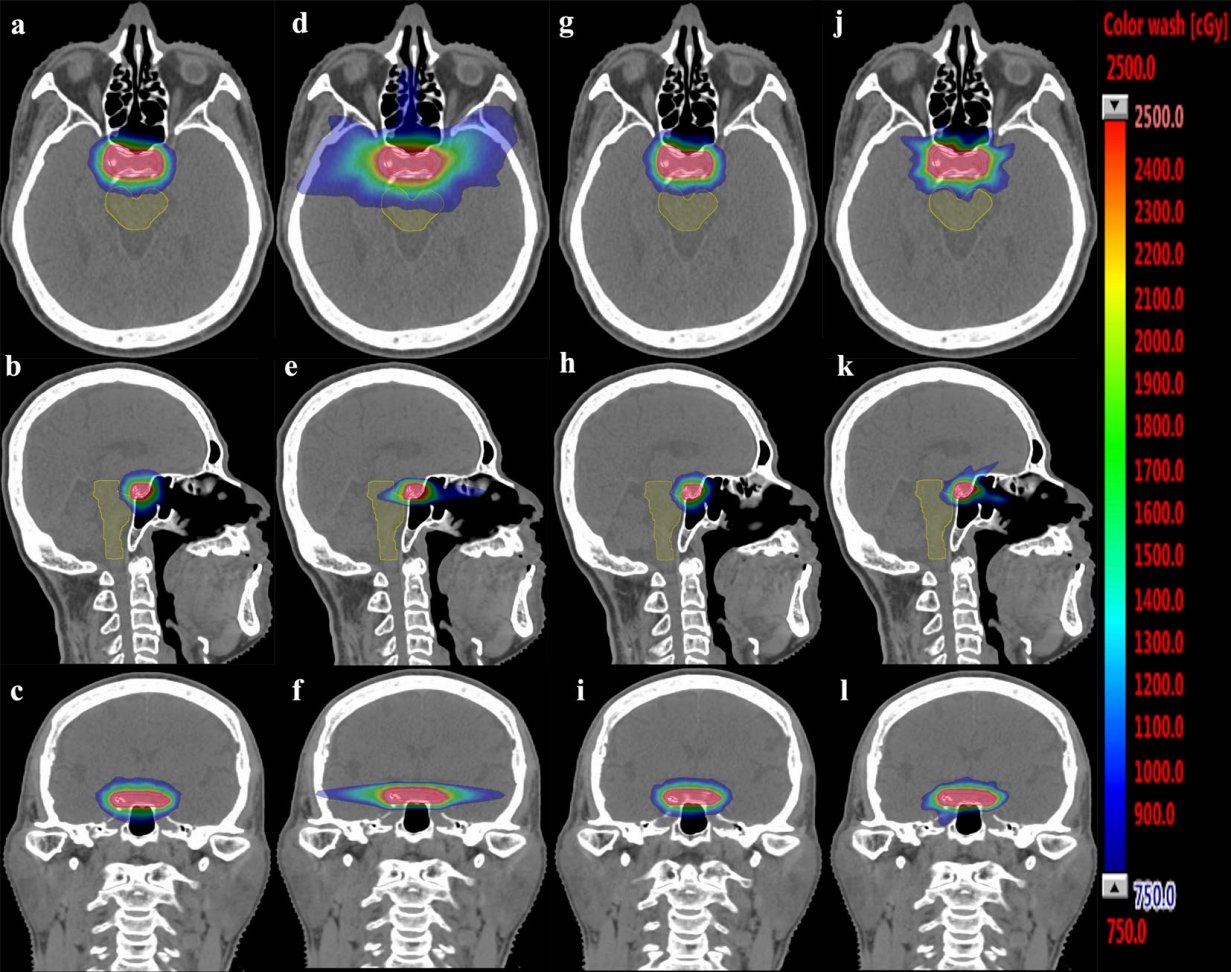
Isodose curves for the applied HyperArc-SRS NTO, RapidArc, HyperArc-ALDO and CyberKnife plans. Clinical target volume: red; planning target volume: pink; brainstem: yellow. (**a–c**) The HyperArc-SRS NTO plan; (**d–f**) the RapidArc plan; (**g–i**) the HyperArc-ALDO plan; (**j–l**) the CyberKnife plan.

**Figure 2:**
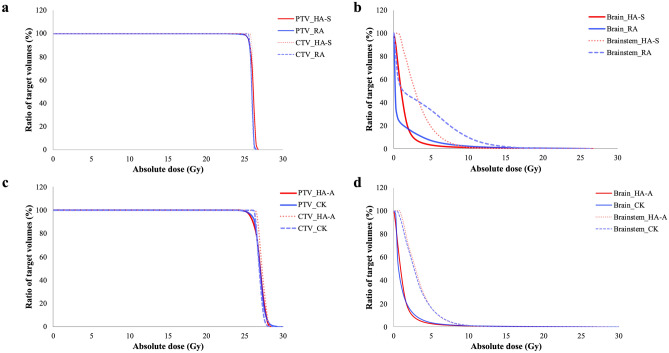
Dose-volume histograms for the applied HyperArc-SRS NTO, RapidArc, HyperArc-ALDO and CyberKnife plans. (**a**) Clinical target volumes (CTV) and planning target volumes (PTV) of the RapidArc and HyperArc-SRS NTO plans; (**b**) brain and brainstem of the RapidArc and HyperArc-SRS NTO plans; (**c**) CTV and PTV of the HyperArc-ALDO and CyberKnife plans; (**d**) brain and brainstem in HyperArc-ALDO and CyberKnife plans; X-axis, the percentage of the prescription dose; Y-axis, the volume percentage of the OAR.

A detailed comparison of dosimetric parameters for the HA-S, coplanar RA, HA-A and cone-based CK plans for the benign brain lesion patients is shown in Table 3. All the constraints for organs at risk (OARs) were met in the HA-S, coplanar RA, HA-A, and cone-based CK plans. The isodose curves and dose-volume histograms (DVH) for the applied HA-S, coplanar RA, HA-A and cone-based CK plans for the patient were presented in Figs. 1 and 2, respectively.

**Table 3:** Comparison of dosimetric parameters for HyperArc, RapidArc, virtual CyberKnife and CyberKnife for the benign brain lesions.

Groups	The homogenous delivery group			The inhomogeneous delivery group		
Parameters	HA-S Mean (SEM)	RA Mean (SEM)	P value HA-S vs. RA	HA-A Mean (SEM)	CK Mean (SEM)	P value HA-A vs. CK
**Target**						
CTV coverage (%)	99.22 (0.34)	98.61 (1.02)	0.767	99.70 (0.19)	98.85 (0.53)	0.008*
D2 (Gy)	26.45 (0.08)	24.86 (1.59)	0.196	28.81 (0.17)	28.72 (0.18)	0.134
D98 (Gy)	25.36 (0.12)	25.13 (0.33)	0.918	26.49 (0.12)	25.83 (0.51)	0.006*
Mean dose (Gy)	25.97 (0.07)	25.97 (0.06)	1.000	27.79 (0.13)	27.72 (0.14)	0.093
PTV coverage (%)	97.64 (0.47)	96.19 (1.09)	0.408	98.70 (0.44)	96.87 (0.67)	0.001*
D2 (Gy)	26.49 (0.08)	26.43 (0.08)	0.234	28.76 (0.17)	28.68 (0.19)	0.326
D98 (Gy)	24.84 (0.14)	24.41 (0.34)	0.438	25.32 (0.26)	24.41 (0.34)	0.030*
Mean dose	25.91(0.06)	25.89 (0.05)	0.501	27.51 (0.11)	27.49(0.11)	0.379
**Organs at risk (Gy)**						
Brainstem (Dmax)	20.83 (1.76)	22.08 (1.50)	0.326	21.23 (1.85)	21.79 (1.79)	0.056
Brainstem (mean)	4.56 (0.51)	6.15 (0.65)	0.001*	4.80 (0.55)	5.08 (0.66)	0.679
Brain (mean)	1.73 (0.23)	1.75 (0.37)	0.109	1.61 (0.12)	1.85 (0.16)	0.005*
Brain V24 (c.c., mean)	6.02 (0.82)	6.77 (0.95)	0.004*	6.09 (0.85)	6.71 (0.96)	0.011*
Brain V12 (c.c., mean)	16.03 (1.90)	28.05 (3.54)	0.001*	14.97 (2.12)	19.88 (2.87)	< 0.001*
Brain V5 (c.c., mean)	61.61 (6.74)	122.69 (12.22)	< 0.001*	57.13 (7.28)	118.51 (22.50)	< 0.001*
Optic nerve_right (Dmax)	11.85 (2.58)	12.92 (2.64)	0.469	13.04 (2.40)	13.35 (2.52)	0.642
Optic nerve_left (Dmax)	10.86 (2.54)	12.15 (2.61)	0.179	11.10 (2.35)	13.28 (2.48)	0.003*
Chiasma (Dmax)	13.58 (2.61)	14.10 (2.48)	0.959	14.92 (2.14)	16.73 (2.01)	< 0.001*
Eye_right (Dmax)	0.43 (0.05)	1.45 (0.38)	0.001*	0.45 (0.07)	2.19 (0.43)	< 0.001*
Eye_left (Dmax)	0.41 (0.05)	0.90 (0.18)	0.006*	0.41 (0.04)	1.96 (0.38)	0.001*
Lens_right (Dmax)	0.17 (0.02)	0.34 (0.09)	0.001*	0.17 (0.02)	0.65 (0.09)	0.001*
Lens_left (Dmax)	0.20 (0.05)	0.28 (0.05)	0.015*	0.16 (0.02)	0.63 (0.09)	< 0.001*
**Dose distribution metrics**						
Conformity index (mean)	0.88 (0.01)	0.80 (0.01)	< 0.001*	0.83 (0.02)	0.79 (0.01)	0.011*
Homogeneity index (mean)	0.06 (0.01)	0.08 (0.01)	0.605	0.12 (0.01)	0.15 (0.02)	0.007*
High dose spillage (mean)	0.01 (0.00)	0.05 (0.03)	0.041*	0.06 (0.01)	0.22 (0.06)	0.005*
Intermediate dose spillage (mean)	3.65 (0.19)	6.06 (0.40)	< 0.001*	3.46 (0.22)	3.93 (0.12)	0.007*
Gradient radius (cm)	0.59 (0.02)	0.88 (0.03)	< 0.001*	0.52 (0.02)	0.63 (0.04)	< 0.001*
Monitor units (mean)	9321 (376)	8426 (640)	0.215	9181 (474)	53,470 (4929)	< 0.001*

*CTV* clinical tumor volume, the volume of the benign brain lesion, *PTV* planning target volume, *SEM* standard error of the mean, *Dmax* the maximal point dose (0.0035 c.c. or less) of the organ at risk, *HA-S* HyperArc technique with SRS NTO optimization, *RA* RapidArc technique, *HA-A* HyperArc technique with ALDO optimization, *CK* CyberKnife technique.

*Statistically significant, *P* < 0.05.

### For homogeneous delivery: noncoplanar HA-S vs. coplanar RA

#### Target coverage

The HA-S and coplanar RA plans produced similar CTV and PTV coverage without any significant difference (CTV: HA-S, 99.22%; coplanar RA, 98.61%; PTV: HA-S, 97.64%; coplanar RA 96.19%).

#### Sparing of organs at risk

Under the same constraints, the mean doses for the brainstem generated by the HA-S plans were significantly lower than those generated by the coplanar RA plans (HA-S plan vs. coplanar RA plan, *P* = 0.001*). The brain V24, V12, and V5, surrogate dose levels for SRS and FRST^18^, were significantly smaller with the HA-S plans than with the coplanar RA plans (V24: 6.02 c.c. in the HA-S plans, 6.77 c.c. in the coplanar RA plans; V12: 16.03 in HA-S; 28.05 in coplanar RA; V5: 61.61 in HA-S; 122.69 in coplanar RA). The maximal doses to the optic nerve and chiasma were similar in the HA-S and the coplanar RA plans. The maximal doses to the bilateral eyes and lens were significantly lower with HA-S plans than with the coplanar RA plans. In Fig. 1, 30% of the prescription isodose line (the prescription dose, 25 Gy) was confined around the target region in the HA-S plan. However, in the coplanar RA plan, 30% of the prescription isodose line was spread out in the coplanar-lateral direction, which might increase the excess doses delivered to OARs, such as the brainstem and brain.

#### Dosimetric parameters

**Figure 3:**
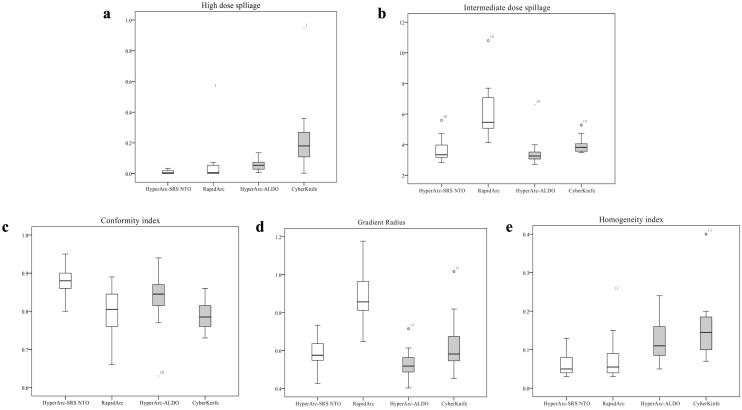
Boxplots of dosimetric parameters HyperArc, RapidArc, HyperArc-ALDO and CyberKnife plans. (**a**) High dose spillage; (**b**) intermediate dose spillage; (**c**) conformity index; (**d**) gradient radius; (**e**) homogeneity index. Boxes, median value and upper and lower quartiles; whiskers, maximum and minimum values within 1.5× interquartile range; dots, outliers.

Figure 3 shows the distributions of the dosimetric parameters of high/intermediate dose spillage, conformity index (CI), gradient radius, and homogeneity index (HI) for the four treatment approaches. The HA-S plans achieved a significantly higher CI (0.88 (HA-S) vs. 0.80 (coplanar RA), P < 0.001) and gradient radius than the coplanar RA plans (0.59 (HA-S) vs. 0.88 (coplanar RA), P < 0.001). In addition, the HA-S plans generated significantly lesser high and intermediate dose spillage than the coplanar RA plans (0.01 (HA-S) vs. 0.05 (coplanar RA), P = 0.041; 3.65 (HA-S) vs. 6.06 (coplanar RA), P < 0.001, respectively). There were no significant differences in high dose spillage or MU production between the HA-S and coplanar RA plans.

### For inhomogeneous delivery: noncoplanar HA-A vs. noncoplanar CK

#### Target coverage

The HA-A and cone-based CK plans exhibited higher coverage of the CTVs, with statistically significant difference (HA-A plans vs. cone-based CK plans, 99.70% vs. 98.85%, P = 0.008); moreover, the HA-A plans exhibited significantly greater PTV coverage than the cone-based CK plans (HA-A plans vs. cone-based CK plans, 98.70% vs. 96.87%, P = 0.001).

#### Sparing of OARs

We analyzed the volume-dose parameters of OAR sparing. All the critical organ-dose requirements were satisfied in the HA-A and cone-based CK treatment plans; however, the HA-A plans consistently achieved better sparing of all OARs than the cone-based CK plans, except for the mean brainstem dose, the maximal dose of the brainstem and right optic nerve, as shown in Table 3. The mean brain doses were 1.61 Gy and 1.85 Gy for HA-A and cone-based CK, respectively (P = 0.005). The brain V24, V12, and V5 were significantly smaller with HA-A plans than with cone-based CK plans (V24: 6.09 c.c. in HA-A vs. 6.71 c.c. in cone-based CK, P = 0.011; V12: 14.97 in HA-A vs. 19.88 in cone-based CK, P < 0.001; V5: 57.134 in HA-A vs. 118.51 in cone-based CK, P < 0.001). The volumes of brain V24, V12, and V5 of the HA-A plans were reduced by 9%, 24%, and 35%, respectively, compared with the cone-based CK plans.

Significant differences in the maximal doses of the optic apparatus (optic nerves, left; optic chiasm; eyes, right and left; lenses, right and left) were observed between the HA-A and cone-based CK plans. As shown in Fig. 1, the 30% prescription isodose line (prescription dose, 25 Gy) was compact to the target in the HA-A plan. The radiation dose in the cone-based CK plan was spiculated and spread out because of the multiple beam angles (200–300 beam nodes).

#### Dosimetric parameters

The HA-A plans, using the ALDO optimization algorithm to improve dose gradients, resulted in all dose distribution metrics (CI, HI, high dose spillage, intermediate dose spillage, and gradient radius) of the HA-A plans were significantly better than those of the cone-based CK plans (P < 0.05) (Fig. 1, Table 3). Moreover, the HA-A plans resulted in 82% fewer MUs than did the cone-based CK plans (HA-A, 9181 MU; cone-based CK, 53470 MU).

## Discussion

Data from published literature indicate that either SRS or FSRT is a crucial component in the management of benign brain lesions with excellent long-term tumor control^27–32^; however, advantages and disadvantages of the different stereotactic fractionations in the management of patients with benign brain lesions and their optimal indications are still a matter of debate. In general, for benign brain lesions, the rationale of SRS or FRST is to control the growth of the lesion without damage to the brain or other OARs; therefore, the prescription dose is relatively lower than malignant lesions, and fractionating the prescription doses make it more tolerable to the OARs. Radiation-induced brain necrosis is one of the common toxicities after the SRS/FSRT treatment. The radiation-induced brain necrosis risk correlated with brain dose/volume metrics. Studies have demonstrated the importance of brain V12 in predicting the necrosis toxicity risks after a SRS treatment^18^. And the use of FSRT appears to reduce the risks of radiation-induced brain necrosis for larger treatment volumes relative to SRS^18^. The dose/volume tolerances of the brain for FSRT, V24 < 20 c.c., was associated with less than 10% risk of any brain necrosis or edema^18^. In this study, we opted to report the results in terms of the volume of brain (included the target volume) receiving absolute levels (5, 12, and 24 Gy) of physical dose, an objective quantity^33^, irrespectively from fractionation.

Intracranial SRS has traditionally been delivered using a cone-based and frame-based SRS platform, but the modern improvement of software and hardware in the MLC-based SRS/FSRT system has made an alternative approach possible^34^. Historically, Linac-based SRS/FSRT has been performed using the static or dynamic conformal arc radiotherapy technique with isocentric irradiation^35,36^. Advances in the VMAT technique in conjunction with the new generation of high-definition MLCs (2.5-mm leaf width) fulfill the demands of SRS dose delivery for small targets within the brain^19^.

Today, the VMAT-based SRS/FSRT technique is a new field of active research. With image-guided, VMAT-based automatic noncoplanar FSRT of benign brain lesions becoming feasible via the newly released HA, it is essential to understand the dosimetric differences among different SRS/FSRT techniques. Various treatment platforms are now available, potentially allowing expansion of services beyond specialist units for easier patient access^27,37,38^. Here, we analyzed the dosimetric results in sixteen patients with a single deep-seated, centrally located benign brain lesions by comparing automatic noncoplanar HA-S with coplanar RA for homogenous delivery and noncoplanar cone-based CK with HA-A for inhomogeneous delivery. Our study was prompted by the lack of data concerning the treatment of the deep-seated, centrally located benign brain lesion using FSRT and the doses received by OARs.

In general, RA can produce a high-quality treatment plan and achieve fast dosing delivery for SRS^12,39^. HD MLC-based Linac treatments deliver doses with isocentric coplanar radiation, resulting in a homogeneous dose distribution and a reduction in low-dose areas^39,40^. In the current study, we demonstrated that the coplanar RA plans consistently achieved adequate target coverages and a low radiation dose to multiple OARs. Under the same hardware conditions, the coplanar HA plans allowed one partial or full coplanar arc and up to 3 noncoplanar arcs in the isocentric plane with new optimization algorithms, SRS NTO. SRS NTO automatically generates virtual shells around the targets to enforce a sharp dose falloff and prevent dose bridging of adjacent disparate targets^13,14^. These differences promoted HA-S dose conformity, intermediate dose spillage, and gradient radius compared with the coplanar RA plans. Another distinguishing advantage of HA-S is consistently reducing the radiation dose to multiple OARs, including dose to the brainstem, brain V24, V12, V5, eyes, and lens, comparing to coplanar RA. Similar findings have been demonstrated that HA has some advantage over RA for intracranial brain metastasis treatment^17,19,22,23^ and extracranial^41^ treatment.

The CK plans in this study were generated for our institution’s fourth-generation CK, which is a cone-based stereotactic radiotherapy system. Cone-based CK repeatedly delivers doses with nonconformal cylinders of radiation, resulting in heterogeneous dose distribution. Whether dose heterogeneity is desirable when homogeneity with sufficient dose conformity can be achieved remains a matter of clinical debate^7,42–44^. The plans for HA-A by ALDO optimization were designed to compare with the inhomogeneous isodose distributions of the cone-based CK. ALDO, the other optimization algorithm for the HA treatment plan that can cover the target with the prescription dose (ideally full coverage) but might create a high inhomogeneous dose within the target to 150% of the prescription dose^17^. Here, we demonstrated that the advantage of HA-A over cone-based CK is that it consistently achieves better dose conformity, high/intermediate dose spillage, dose gradients, and sparing of all OARs. As a result of the improved CI and dose gradients of the HA-A plans compared to the cone-based CK plans, significantly improved target coverage was achieved using the HA-A plan without compromising the sparing of OARs. Therefore, HA-A can produce a high-quality treatment plan with excellent target coverages and dosimetric parameters under inhomogeneous delivery.

A vital factor for successful SRS/FSRT treatment of benign brain tumors is the quality of the treatment plan. Currently, improvements in hardware and software have made the quality of the HA treatment plan higher than ever. HD-MLC-based noncoplanar VAMT and the usage of flattening-filter-free beams allow high dose conformity and fast dose falloff delivery^40,45^. The spreading of the radiation dose is the nature of noncoplanar delivery. At the very least, more entrance and exit doses from the noncoplanar arcs would lead to more low-dose regions.

The HA plans optimized the collimator angle for each arc and used jaw tracking^46^ to prevent excessive radiation doses from reaching the normal brain, and other OARs due to leakage and transmission through the MLC leaves. During the HA treatment plan optimization process, the application of the SRS NTO is novel and was designed to produce the most compact dose falloff possible, featuring steep spatial dose decay from target-specific dose levels to low asymptotic dose levels resulting in reduced OAR exposure and minimal interaction between targets^19^. The application of ALDO to HA maintains the similar dosimetric features of SRS NTO but more focusing on creating very high target coverage with acceptable inhomogeneous doses within the target^17^. These HA advances result in very effective control of high and intermediate dose spreading while maintaining tumor coverage and dose conformity.

To our knowledge, no study of HA for benign brain lesions has compared it with other SRS/FSRT techniques. Some studies have been conducted for dosimetric analysis of multiple brain metastases. Kadoya et al. reported that the HA plans are comparable to the CK plans for multiple brain metastases^23^. In general, there are no significant differences between the CK and HA plans from the physicians’ viewpoint. However, the HA plans had better CI and brain V12 than the CK plans, while the CK plans showed better GI than the HA plans^23^. Slosarek et al. demonstrated that the use of HA ccould significantly improve the sparing of the healthy brain while maintaining full coverage of the target volumes, comparing to VMAT and CK in silico study^33^. Thomas et al. demonstrated that noncoplanar VMAT, the former HA model, produces clinically equivalent conformity, dose falloff, brain V12, and low isodose spillage to a gamma knife system^47^, along with shorter treatment time.

The current study still has some limitations. The first limitation of this study was that HA plans with highly precise mechanical geometry and a brand-new optimization schema were compared to routine clinical CK plans that had previously been delivered; this was an unavoidable feature of the study design. Our ultimate intent is to know whether we could use the HA system to replicate the high-quality plan that we were already achieving with CK and determine the limits of the HA technique.

Second, delivery at a Linac may require an increased PTV margin to account for delivery and setup errors. For the HA plans, we did not modify the contours of the targets and OARs, including the PTV margin. The current CK G4 system can achieve submillimeter (< 1 mm) accuracy and precision^48^. With the improved technology, modern Linacs, such as the TrueBeam system, can achieve overall couch/gantry/collimator isocentric accuracy within 0.6–0.75 mm^49,50^. When they are combined with image guidance capabilities using CBCT, a targeting accuracy of 0.5 × 0.2 mm can be achieved for small intracranial targets^51^. Due to the similar submillimeter accuracy of TrueBeam and CK, the same corresponding PTV margin does not seem to be a major concern in delivery accuracy or dose distribution.

The third limitation of our study was that the CK M6 system was unavailable for this dosimetric comparison. The new generation of the CK M6 system using MLC has been shown to improve the treatment plan quality^52^ based on the G4 accuracy^48^. The CK-MLC plans generated for the SRS treatment delivered a more homogeneous dose to the target than the cone-based CK plans, providing equivalent coverage, conformity, and OAR doses with the potential to improve treatment quality—for example, by achieving a better dose gradient in the low-dose region^53–55^. The new CK system's primary advantages are a 30–35% reduction in treatment time and a reduction in MU compared to the cone-based CK plans^53–55^. The median treatment time per fraction in the fifteen enrolled patients treated with CK was 75 min. Although the delivery time of the new CK (VSI and M6) system has been improved (25–30 min per fraction)^52^, owing to the step-and-shoot method and the travel time of the robot between delivery nodes, treatment efficiency may still be a drawback compared to the continuous arc delivery provided Linac-based systems^52^. Moreover, one of the novel features of HA is automated delivery workflow; in general, the average time spent on HA treatment of the first forty patients at our institution is 13.23 min, which is very close to the time slot (10 min) allotted for conventional fractionation in our institution.

In short, adequate sparing of OARs, proper dosimetric distribution, accuracy, and fast delivery make HA an attractive FSRT technique for deep-seated benign brain tumors. Further clinical studies using HA for benign brain lesions would be necessary to determine the long-term tumor control rates and the radiation-induced toxicity profiles following SRS or FSRT.
